# Correlation among genetic, Euclidean, temporal, and herd ownership distances of porcine reproductive and respiratory syndrome virus strains in Quebec, Canada

**DOI:** 10.1186/1746-6148-8-76

**Published:** 2012-06-07

**Authors:** Marie-Ève Lambert, Julie Arsenault, Zvonimir Poljak, Sylvie D’Allaire

**Affiliations:** 1 Faculty of Veterinary Medicine, University of Montreal, St. Hyacinthe, Quebec, Canada; 2 Department of Population Medicine, Ontario Veterinary College, University of Guelph, Ontario, Canada

**Keywords:** Porcine reproductive and respiratory syndrome virus (PRRSV), ORF5 sequences, Genetic distances, Mantel test, Correlation

## Abstract

**Background:**

Porcine reproductive and respiratory syndrome (PRRS) is a viral disease that has a major economic impact for the swine industry. Its control is mostly directed towards preventing its spread which requires a better understanding of the mechanisms of transmission of the virus between herds. The objectives of this study were to describe the genetic diversity and to assess the correlation among genetic, Euclidean and temporal distances and ownership to better understand pathways of transmission.

**Results:**

A cross-sectional study was conducted on sites located in a high density area of swine production in Quebec. Geographical coordinates (longitude/latitude), date of submission and ownership were obtained for each site. ORF5 sequencing was attempted on PRRSV positive sites. Proportion of pairwise combinations of strains having ≥98% genetic homology were analysed according to Euclidean distances and ownership. Correlations between genetic, Euclidean and temporal distances and ownership were assessed using Mantel tests on continuous and binary matrices. Sensitivity of the correlations between genetic and Euclidean as well as temporal distances was evaluated for different Euclidean and temporal distance thresholds. An ORF5 sequence was identified for 132 of the 176 (75%) PRRSV positive sites; 122 were wild-type strains. The mean (min-max) genetic, Euclidean and temporal pairwise distances were 11.6% (0–18.7), 15.0 km (0.04-45.7) and 218 days (0–852), respectively**.** Significant positive correlations were observed between genetic and ownership, genetic and Euclidean and between genetic and temporal binary distances. The relationship between genetic and ownership suggests either common sources of animals or semen, employees, technical services or vehicles, whereas that between genetic and Euclidean binary distances is compatible with area spread of the virus. The latter correlation was observed only up to 5 km.

**Conclusions:**

This study suggests that transmission of PRRSV is likely to occur between sites belonging to the same owner or through area spread within a 5 km distance. Both should be considered in the perspective of prevention.

## Background

Porcine reproductive and respiratory syndrome (PRRS) is a viral disease representing one of the most challenging threats to swine industry. This disease has a major economic impact, inducing late-term abortions, stillbirths, mummified and weak piglets in breeding herds and respiratory disease, increased mortality rate and poor growing performances in growing pigs [1,2].

PRRS is caused by a spherical, enveloped, single-stranded and positive-sense RNA virus belonging to the family of *Arteriviridae* within the genus *Arterivirus* and the order *Nidovirales*[3]. An important genetic and antigenic diversity of PRRSV strains is reported within and among the European (EU) and North American (NA) genotypes [4,5]. This extensive genetic diversity seriously impairs PRRSV management, as only partial protective immune response is obtained after experimental heterologous challenge [6-8]. Both modified-live vaccines available at the time of the study were each based on a single PRRSV strain. Consequently, control of PRRSV is mostly directed towards preventing its spread which requires a better understanding of the mechanisms of transmission of the virus between herds.

The virus can be transmitted between herds through several mechanisms, including introduction of infected animals or semen [9-14]. Contaminated vehicles can also serve as mechanical vectors to transmit the virus as they can convey the virus on significant distances on their wheels [15,16]. The proximity or high density of pig sites in neighbourhood has been recognized as a risk factor for PRRSV, which might involved a local transmission by aerosols, insects and avian species [10,17-22]. Fomites as boots, coveralls or other equipments conveyed by people can contribute as well to area spread of PRRSV [17,23,24].

Based on the premise that PRRSV strains sharing a high level of homology is suggestive of a common source of infection, molecular epidemiology can help improving our understanding of mechanisms of PRRSV dispersion over space and time [25,26]. The objectives of this study were to describe the genetic diversity observed for PRRSV according to some characteristics of production sites and to assess the correlation between genetic, Euclidean and temporal distances and ownership to suggest different pathways for PRRSV transmission in a high density area of swine production.

## Methods

### Study design, data collection and laboratory analyses

A cross-sectional study was conducted in the Monteregie administrative region in the province of Quebec, Canada, between February 2005 and June 2007. Within this region, a high density area (HD) was purposely targeted for a larger project on transmission and control of PRRSV, and corresponded to 10 adjacent municipalities covering approximately a 20 km-radius area [27] with 354 pigs/km^2^[28]. A site was defined as one or more units located within 300 m from another, belonging to the same owner (individual or corporate), and having the same animal source(s). In order to select all sites located in the HD area, all producers listed into the Quebec Federation of Pork Producers (FPPQ) database were contacted. Participation was obtained through a voluntary basis. The participation percentage among contacted producers for the larger project was 77% [27]. All sites of each participating producer were included in the study. Data were gathered on different herd and neighbourhood characteristics of the sites and geographical coordinates (latitude/longitude) were obtained using global positioning system (GPS). Among participating sites, 176 were considered as PRRSV positive (see [29] for methodological details). For those sites, open reading frame 5 (ORF5) sequencing was attempted on tissue (pooled lungs, tonsils, and tracheobronchial lymph nodes) or sera, depending on clinical history of the herd [29]. PCR products were purified before sequencing with Qiaquick spin kit (Qiagen). Sequencing of ORF5 was done on both directions of PCR products using amplification primers with BigDye terminator on ABI PRISM 310 Genetic analyzer (Applied Biosystems Canada, Streetsville, Ontario, Canada). All diagnostic procedures were done at the Faculty of Veterinary Medicine of the University of Montreal in St. Hyacinthe. The procedures were approved by the Comité d’éthique de l’utilisation des animaux of the University of Montreal (certificate number: 09-rech-1291).

### Statistical analyses

ORF5 sequences having ≥98% pairwise homology with MLV (Ingelvac® PRRS MLV or ReproCyc® PRRS-PLE, Boehringer Ingelheim, (Canada) Ltd.) or ATP (Ingelvac® PRRS ATP, Boehringer Ingelheim, (Canada) Ltd.) commercial vaccines strains were excluded from further analyses whereas the remaining sequences were considered as wild-type strains.

#### Calculation of various distances

For each pair of sites, the genetic, Euclidean, temporal and ownership distances were calculated. Pairwise genetic distances were defined as the percentage of homologous nucleotides between sequences. It was calculated from nucleotides using Juke and Cantor substitution model in Bionumerics software (Applied Maths Inc., version 6.5, Austin, TX, USA), following a pairwise alignment of ORF5 sequences. For the Euclidean distances, geographical coordinates (latitude/longitude) of the sites were transformed in metric units using Quebec Lamber Conic Conform projection in ArcInfo (Esri, version 9.3, Redlands, CA, USA). Pairwise Euclidean distances between sites were then calculated in SAS (SAS Institute Inc. version 9.1, Cary, NC, USA) using the projected coordinates [30]. Pairwise temporal distances were defined as the number of days separating the sampling of strains. Pairs of sites were classified as having the same ownership if they were from the same independent producer or integrated production system, and as having different ownership otherwise.

#### Descriptive statistics

Descriptive statistics on herd characteristics were performed in SAS. Means of genetic, Euclidean and temporal pairwise continuous distances were calculated. Number of pairwise combinations of strains having ≥98% homology over total number of combinations was computed according to different Euclidean distance thresholds (≤5 km, 5 to ≤10 km, >10 km) and ownership (same vs. different). The Euclidean distance thresholds were defined according to the literature. Most mechanisms of transmission acting on a local scale as aerosols, insects, mammalian species and fomites are more likely to occur at a distance of less than 5 km, whereas transmission occurring at more than 10 km apparently necessitates human interventions, since infectious virus was not identified in aerosols at more than 9.1 km from a population source [22].

#### Correlation analysis

Bivariate Mantel tests for genetic, Euclidean, temporal continuous distances and ownership were computed in R (R Foundation for Statistical Computing, version 2.9, Vienna, Austria) using vegan package and Pearson correlation coefficient [31]. Bivariate Mantel tests were also computed to assess the correlation between genetic, Euclidean, temporal binary distances and ownership. Dichotomization was performed as follows: genetic distance (≥98% vs. <98% homology), Euclidean distance (≤5 vs. >5 km), temporal distance (≤1 vs. >1 month), and ownership (same vs. different). Genetic homology threshold for similar strains (≥98%) was chosen according to a previous molecular epidemiological study [11]. On binary matrices, significant correlations with genetic distance were also evaluated after adjustment for other distances using partial Mantel test. Correlations having P-value ≤0.05 after 9999 permutations of matrices were considered significant. In view of the discrepancy of the correlations observed between genetic and Euclidean distances when using continuous or binary matrices, the sensitivity of results to the selection of different thresholds was further investigated. The bivariate correlation between genetic and Euclidean binary distances and between genetic and temporal binary distances was examined using Mantel test for thresholds ranging from 1 to 20 km by km and from 1 to 12 months by month, respectively. Results were presented into correlograms made in R. For each correlogram, the level of significance for individual tests was adjusted using Bonferroni procedure to account for multiple testing to obtain a family level of significance of α = 0.05 [32].

## Results

An ORF5 sequence was identified for 132 out of the 176 (75%) infected sites. ORF5 sequences having ≥98% homology with MLV and ATP vaccines strains were observed for seven and three sites, respectively. On these latter sites, the use of commercial vaccination was confirmed or highly suspected. Consequently, 10 sites composed of two farrow-to-finish and eight weaners and/or finishers operations were excluded from further analyses, the remaining 122 sequences being considered as wild-type PRRSV strains. Table 1 described herd and neighbourhood characteristics of sites (n = 122). Sites were managed by an independent producer or by an integrated company on 65% and 35% of the sites, respectively. The 43 integrated sites belonged to 8 companies, with one owning 53% of these sites. Independently owned sites (n = 79) were managed by 60 different producers; 45, 11 and 4 producers managing 1, 2 or 3 sites, respectively. Production sites were attended by 23 different veterinarians.

**Table 1:** Characteristics of sites (n = 122) where PRRSV wild-type ORF5 sequence was identified according to production type

**Characteristics of sites**		**Breeding sites**	**Growing sites**
		n = 53	n = 69
**Categorical variables**		**%**	**%**
Production type			
	Farrowing-to-wean	15	-
	Farrow-to-grow	6	-
	Farrow-to-finish	79	-
	Weaners	-	16
	Weaner-to-finish	-	14
	Finishers	-	70
Ownership			
	Independent producer	92	43
	Contract producer	8	57
Distance from public road			
	>300 m	6	22
	≤300 m	94	78
**Continuous variables**			
Number of productive sows	median (Q1-Q3)	185 (136–300)	-
Total number of animals^a^	median (Q1-Q3)	1350 (891–2020)	1550 (1000–2450)
Distance from closest pig site (m)^b^	median (Q1-Q3)	400 (220–610)	409 (200–1000)

^a^ Including gilts, sows, weaners and finishers if present on site, ^b^ Approximated by the producer.

All wild-type ORF5 sequences belonged to the North American genotype and most sequences (92%) had a length of 603 bp with the exception of 7 strains presenting insertion (606 bp) or 3-base deletions (600 bp). The mean (min-max) genetic, Euclidean and temporal pairwise distances were 11.6% (0–18.7), 15.0 km (0.04-45.7) and 218 days (0–852), respectively. Among the 122 wild-type PRRSV strains, 62 (51%) did not show any ≥98% pairwise genetic homology with other (s) sequence (s), 34 (28%) showed this latter homology with one strain only and 26 (21%) with more than one strain. Using dichotomized distances, proportion of pairwise combinations of strains sharing ≥98% homology over total number of combinations is reported in Table 2 according to ownership (same vs. different) and Euclidean distance using different thresholds (≤5 km, >5 to ≤10 km, >10 km).

**Table 2:** Number of pairwise combinations with ≥98% genetic homology for PRRSV ORF5 over total number of combinations (%) according to Euclidean distance between sites and herd ownership (122 sequences)

**Euclidean distance**		**Ownership**		**Total**
		**Same**	**Different**	
	**≤5 km**	8/50 (16.0%)	7/785 (0.9%)	15/835 (1.8%)
	**>5 to ≤10 km**	8/83 (9.6%)	5/1453 (0.3%)	13/1536 (0.8%)
	**>10 km**	9/174 (5.2%)	20/4853 (0.4%)	29/5010 (0.6%)
	**Total**	25/307 (8.1%)	32/7074 (0.5%)	57/7381 (0.8%)

Bivariate correlations between genetic, Euclidean and temporal continuous distances were computed on the 122 PRRSV wild-type strains using 7381 different pairwise combinations. Results are shown in Table 3. No significant correlation was observed between genetic and Euclidean continuous distances and between Euclidean and temporal continuous distances (P >0.05). However, positive correlations were identified between genetic and temporal continuous distances and between genetic continuous distance and ownership (P ≤0.01). Bivariate correlations obtained using genetic (≥98%, <98% homology), Euclidean (≤5, >5 km), temporal (≤1, >1 month) binary distances and ownership (same, different) are also presented in Table 3. Significant correlations involving genetic distances were marginally influenced by adjustment for anyone of the other binary distances using the Partial Mantel Test (Table 4).

**Table 3:** Bivariate correlations between genetic, Euclidean and temporal distances and herd ownership computed with Mantel test procedure (122 PRRSV ORF5 sequences)

**Distance variable 1**	**Distance variable 2**	**r**_**M**_^**a**^	**P-value**^**b**^
Genetic^c^	Ownership^d^	0.07	0.01
Genetic^c^	Euclidean^c^	−0.008	0.56
Genetic^c^	Temporal^c^	0.11	<0.01
Euclidean^c^	Temporal^c^	−0.009	0.58
Genetic^e^	Ownership^d^	0.17	<0.01
Genetic^e^	Euclidean^f^	0.04	<0.01
Genetic^e^	Temporal^g^	0.06	<0.01
Temporal^g^	Euclidean^f^	0.02	0.05
Temporal^g^	Ownership^d^	0.05	<0.01
Euclidean^f^	Ownership^d^	0.03	0.03

^a^ Mantel R test statistic using a Pearson correlation coefficient, ^b^ P-value obtained with 9999 Monte Carlo simulations, ^c^ Continuous distance, ^d^ 0: same, 1: different, ^e^ 0: ≥98%, 1: <98% homology, ^f^ 0: ≤5 km, 1: >5 km, ^g^ 0: ≤1 month, 1: >1 month.

**Table 4:** Correlations between genetic, Euclidean and temporal binary distances and ownership computed with Partial Mantel test procedure (122 PRRSV ORF5 sequences)

**Distance variable 1**	**Distance variable 2**	**Distance variable 3**	**r**_**M**_^**a**^	**P-value**^**b**^
Genetic^c^	Euclidean^d^	Ownership^e^	0.04	<0.01
		Temporal^f^	0.04	0.02
Genetic^c^	Ownership^e^	Euclidean^d^	0.17	<0.01
		Temporal^f^	0.17	<0.01
Genetic^c^	Temporal^f^	Euclidean^d^	0.06	<0.01
		Ownership^e^	0.05	<0.01

^a^ Partial Mantel R test statistic using a Pearson correlation coefficient, ^b^ P-value obtained with 9999 Monte Carlo simulations, ^c^ 0: ≥98%, 1: <98% homology, ^d^ 0: ≤5 km, 1: >5 km, ^e^ 0: same, 1: different, ^f^ 0: ≤1 month, 1: >1 month.

**Figure 1:**
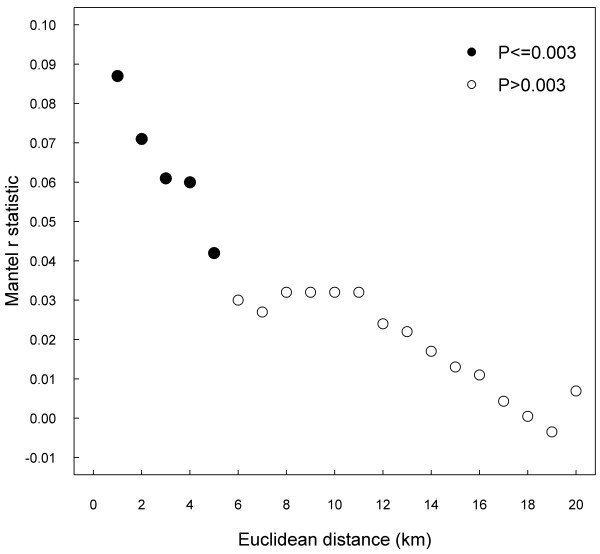
**Correlogram showing Mantel r statistic computed on binary matrices of genetic (≥98%, <98% homology) and Euclidean distances for different thresholds (km).** Results from 7381 different pairwise combinations of 122 PRRSV ORF5 sequences. Dark dots indicate significant correlation (P ≤0.003) after 9999 permutations of matrices.

**Figure 2:**
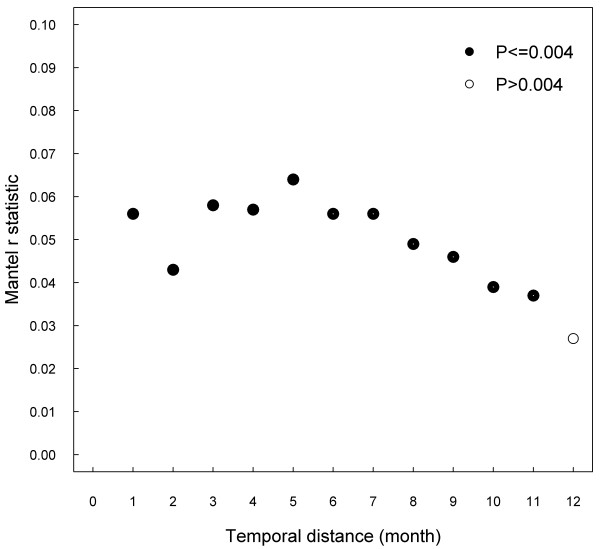
**Correlogram showing Mantel r statistic computed on binary matrices of genetic (≥98%, <98% homology) and temporal distances for different thresholds (month).** Results from 7381 different pairwise combinations of 122 PRRSV ORF5 sequences. Dark dots indicate significant correlation (P ≤ 0.004) after 9999 permutations of matrices.

Figure 1 shows a spatial correlogram of Mantel test statistic (r_m_) obtained between binary matrices of genetic and Euclidean distances for different Euclidean thresholds. Significant positive correlations were observed up to 5 km, whereas non significant correlations were noted for any other higher thresholds (up to 40 km). Positive correlations between genetic and temporal binary distances were observed for 11 months, being not significant thereafter (Figure 2).

## Discussion

The mean pairwise genetic distance among PRRSV strains was considerable (11.6%), which is almost the double of the 6.5% genetic diversity obtained for 55 sequences submitted by 48 farms from the Midwest of United States [26]. The maximum genetic distance also exceeded the 12% mentioned in another report using sequences from 62 individual farms belonging to a single pork-producing company in South Dakota [25]. These latter studies were conducted on a small number of sites and it was not reported whether vaccine-like strains were included in their computations which could have contributed to lower the mean and extent of genetic diversity. Surprisingly, for the small geographical and temporal frames, our results were rather closed to the 12.5% average pairwise diversity obtained in phylogenetic analyses of 8624 ORF5 sequences gathered world-wide from the North American, Asian and European continents and over more than 15 years [33]. The extent of genetic diversity certainly adds to the complexity of PRRSV management [6]. However, its impact on PRRSV regional control feasibility is difficult to assess since previous initiatives did not report such diversity within their targeted zone [34-36].

A correlation between genetic distance and ownership was observed, which to the best of our knowledge has never been explored before. About 16 times more pairwise combinations of strains sharing ≥98% homology were observed for sites having the same owner compared to those having different owners (Table 2). Results were also corroborated by the Mantel tests showing that sites managed by same owner globally had more often ≥98% homology, even when adjusting for Euclidean distance (Tables 3 and 4). It suggests a common source of animals or semen [9-11]. Common employees or personnel from technical services could also convey the virus between sites on different fomites such as boots, coveralls or vehicles [15,23,24]. As the exact source of pigs for each site was not available, the variable ownership was our best asset to examine the correlations but it resulted in difficulties in identifying precisely the mechanism(s) involved behind the concept of ownership. For independent producers, multi-site production system designed for early segregated weaning [37] would fit the assumption of a common animal source. In contrast, the use of ownership oversimplified the pyramidal structure of integrated systems.

Similarly to other results [26], we did not observe any correlation between genetic and Euclidean distance with continuous matrices. However, a significant correlation was found with binary distances. As the Mantel test is based on a linear correlation, a nonlinear relationship may be lost using that technique [38]. Therefore, a significant correlation limited to a particular scale might be diluted by inclusion of other distances leading to an overall absence of relationship. With binary distances, two to three times more combinations of strains having ≥98% homology were observed in pairs of sites located at ≤5 km from each other compared to others with distances >5 km, and this association was supported by the Mantel using binary distances (Tables 2,3 and 4). Area spread which is possibly involved behind this relationship represents between-herd transmission occurring without any pig contact, but rather through several pathways of transmission: aerosols (up to 9.1 km), houseflies (up to 2.3 km), mosquitoes and potentially, other mammalian or avian mechanical vectors [11,20-22,39-41]. Vehicles and inert fomites such as boots, coveralls or other equipments conveyed by people combined with absence of biosecurity measures could contribute as well to area spread of PRRSV [15,17,24]. Area spread has been frequently suspected among herds having similar strains in Quebec as well as in different states in United States [11,25,39]. Results from sensitivity analysis supported the 5 km threshold previously chosen; correlation between genetic and Euclidean distances decreasing rapidly and almost linearly up to 5 km, to become not significant thereafter (Figure 1). Virus survival might explain the decrease of the strength as greater Euclidean distances favour longer exposure to adverse environmental conditions. Vulnerability of PRRSV to ultraviolet light, level of humidity and high temperature is demonstrated [42-44].

The positive correlation observed between genetic and temporal distances is compatible with genetic evolution of the virus in the area over a two year period of observation and was also reported by others [26]. Interestingly, when examining the temporal correlogram (Figure 2), the relationship was more linear than the one for genetic and Euclidean distances, which might explain that correlations were significant for both continuous and binary matrices of distances. According to the ability of the virus to mutate over time and considering that new viruses can be introduced into the area through animal sources or transportation, a certain level of evolution of the viruses present in the area is expected. The correlation between genetic and time decreased slowly and was observed up to 11 months, indicating that virus populations are changing over time but that certain viruses might persist on the territory over about 1 year. The time frame for which sequences were obtained was quite large. It could partially explain the low frequency of homologous PRRSV strains observed on the territory. Indeed, compared to short time interval between sampling time, large intervals could have lead to an increased possibility of new virus introduction or to mutation of the virus within herds. Because of the cross-sectional design, the sampling time did not necessarily correspond to the moment of virus introduction on the site and consequently the real temporal process of transmission between farms could not be assessed.

This study attempted to include all sites located in a restricted geographical scale to obtain more precision in exploring area spread compared to other studies [25,26]. However, some producers did not want to participate in the survey whereas others could not be contacted. The fairly good participation rate (77%) improved the internal validity of the study, but total absence of selection bias was impossible to assess. Moreover, underestimation of our participation is possible as some unreached producers might have been out of business. Even if the sampling strategy used on sites with PRRS history was performed in order to maximize the probability of identifying a PRRSV strain, it was not possible to identify a sequence for 25% of the PRRSV positive sites. Whether the exclusion of the latter sites influences the estimate of genetic diversity cannot be determined. Furthermore, only one PRRSV strain was identified per site, assuming the existence of a sole viral strain or at least obtaining the predominant strain. Although not frequently reported, more than one strain can co-exist on a site [11,45]. Analyses were performed using ORF5 gene based on its high genomic variability and its widely use for molecular epidemiology studies on North American PRRSV strains or in studies assessing the extent of genetic diversity [25,46,47]. However, ORF5 gene is only a part of the whole 15 000 kb genome [48] and it could be interesting to compare results when using the entire genome rather than just ORF5 for this kind of analyses. Also, more than 50% of strains gathered in this restricted geographic area did not show any homology of ≥98% with other strains. These findings suggest that additional investigations using different geographical boundaries would allow the study of other mechanisms of transmission acting on a larger scale and involving human intervention such as pig transportation.

## Conclusions

The study brought useful information regarding PRRSV epidemiology in a perspective of prevention. Results support the concept of area spread and also highlighted the role of ownership in viral transmission through different potential mechanisms such as common personnel, vehicles and source of animals.

## Competing interests

The authors declare that they have no competing interests.

## Authors’ contributions

MEL participated in the design of the study, collected all data, performed the statistical analyses, interpreted data and wrote the initial draft of the manuscript. JA provided valuable expertise and support on statistical analyses, participated in data interpretation and critically reviewed the manuscript. ZP provided valuable expertise and support on statistical analyses, and participated in data interpretation. SD participated in the design of the study and data interpretation and helped to draft the manuscript. All authors read and approved the final manuscript.
